# Black Soldier Fly Oil in Different Lipid Diets Could Regulate Tissue Lipid Metabolism and Fatty Acid Composition of Juvenile Mirror Carp

**DOI:** 10.1155/2024/8718694

**Published:** 2024-03-20

**Authors:** Xinxin Xu, Beibei Ji, Ronghua Lu, Hong Ji

**Affiliations:** ^1^College of Fisheries, Henan Normal University, Xinxiang 453000, China; ^2^College of Life Sciences, Henan Normal University, Xinxiang 453000, China; ^3^College of Animal Science and Technology, Northwest A&F University, Yangling, 712100, Shaanxi, China

## Abstract

In the present study, our aim was to assess the effect of dietary black soldier fly oil (BSFO) with different lipid contents on the growth performance, body composition, lipid metabolism, and related gene expression in juvenile mirror carp (*Cyprinus carpio* var. specularis). An 8-week feeding trial was conducted using four isonitrogenous diets (32.0% crude protein) containing two lipid levels: 6% (control (CT) group) and 9% (high lipid (HL) group), both using soybean oil as the oil source. The other two diets contained BSFO on the basis of 6% and 9% lipid, referred to as (CT + BSFO) and (HL + BSFO) groups. The results showed that final body weight, weight gain rate, specific growth rate, protein retention ratio, and feed utilization were significantly increased by using higher dietary lipid levels (*P* < 0.05). Additionally, the gene expression of lipid synthesis markers was significantly up-regulated in intra-peritoneal fat but significantly down-regulated in the hepatopancreas due to increased dietary lipid level (*P* < 0.05). No negative effects on feed utilization or growth performance were observed in fish fed diets containing BSFO. However, the intra-peritoneal fat index, adipocyte size, and hepatopancreas lipid content were significantly decreased in the CT + BSFO group compared to the CT group (*P* < 0.05). Furthermore, BSFO also up-regulated the expression of lipid lipolysis markers in the hepatopancreas and intra-peritoneal fat (*P* < 0.05). Moreover, the use of BSFO also increased the content of n-3 polyunsaturated fatty acid (PUFA) while reducing the content of n-6 PUFA in the muscle (*P* < 0.05). In conclusion, HL diets improved the growth of the fish and increased the lipid deposition. BSFO in the diet resulted in a reduction of lipid deposition in the hepatopancreas and intra-peritoneal fat, likely due to an increase in lipid oxidation.

## 1. Introduction

Dietary lipid plays an important role in the growth and development of fish, as it promotes the absorption of lipophilic nutrients and provides energy and essential fatty acids [1]. Mirror carp (*Cyprinus carpio* var. specularis), a common carp species, is widely farmed in China due to its taste, high nutritional value, and affordable price [2]. It is generally considered that the optimal dietary lipid content for common carp is around 50–60 g kg^−1^ [3]. However, with the development of the intensive breeding industry, high-lipid diets are often used in aquatic production to meet the demands of rapid growth [4]. Increasing the dietary lipid level is a common strategy to promote fish growth and effectively spare dietary protein in many fish species [5, 6]. However, some studies indicate that high-lipid diets may lead to some negative effects, including excessive fat deposition, abnormal oxidative status, and even a decrease in nutritional value [7–9].

Although most of the research on black soldier fly (BSF) as an aqua-feed ingredient has focused on the use of BSF larvae meal [10], black soldier fly oil (BSFO) is gaining increasing attention [11–13]. BSFO is interesting because it differs from most other insect and even plant oils in its fatty acid (FA) composition containing a large amount of the medium-chain fatty acids (MCFAs), for example, lauric acid (C12 : 0) typically represents 21%–50% of the total fatty acids (TFAs) [14, 15]. The uptake and transport of medium and short-chained FAs are quite different from the longer-chained FAs commonly found in traditional lipids of the sense [16]. Lauric acid may act like a direct source of energy, similar to sugar, than a lipid in the more traditional sense. A study by Li et al. [14] demonstrated that replacing soybean oil (SO) with BSFO (25 g kg^−1^ diet) reduced intra-peritoneal fat deposition and decreased adipocyte size in juvenile Jian carp. Xu et al. [13] also showed that BSFO (25 g kg^−1^ diet) had similar effects on lipid metabolism in juvenile mirror carp when compared to silkworm or yellow mealworm oils (YMOs). However, to our knowledge, little is known about the effects of BSFO on improving growth, lipid metabolism, and health status in fish fed diets with different lipid levels. The unique uptake and oxidation of lauric acid compared to other FAs commonly included in carp aqua-feeds may affect the tolerance to higher lipid levels when including BSFO. Therefore, this study was aimed to investigate the effect of high-lipid diets on juvenile mirror carp and whether BSFO could enhance the utilization of such diets, based on growth performance, FA composition, morphology observation, lipid metabolism, and related gene expressions in juvenile mirror carp.

## 2. Materials and Methods

### 2.1. Experimental Diets and Design

In this study, a 2 × 2 factorial design was used. Four isonitrogenous diets (320 g kg^−1^ crude protein) were formulated with two lipid sources (SO or BSFO) at two different lipid levels (60 or 90 g kg^−1^ crude lipid). Two diets were prepared with lipid levels of 60 and 90 g kg^−1^, using 25 g kg^−1^ SO as the base oil source. These diets were named the control (CT) group and high lipid (HL) group, respectively. Additionally, the diets in the CT and HL groups, where the SO was replaced by 25 g kg^−1^ BSFO, were named CT + BSFO and HL + BSFO, respectively.

BSF fed by wheat bran was obtained from Ankang Fisheries Experimental and Demonstration Station of Northwest A&F University (Ankang, Shaanxi Province, China). BSFO was obtained with an oil press machine at a temperature ranging between 80 and 90°C and a pressure between 5 and 20 KP (Dongdubao Electrical Technology Limited Company, Yueqing City, Zhejiang Province, China). The other feed ingredients were provided by Huaqin feed factory (Yangling, Shaanxi Province, China). The experimental diets were formulated with the 2.5 mm pellet diameter and collected after drying in a cool and well-ventilated place at room temperature for 24 hr in Ankang Fisheries Experimental and Demonstration Station of Northwest A&F University. Then, the diets were stored at −20°C until use.

### 2.2. Feeding Trial

Juvenile mirror carp were provided by Ankang Fisheries Experimental and Demonstration Station of Northwest A&F University. Fish were acclimated to the indoor rearing conditions for 2 weeks while being fed commercial diets (Huaqin feed factory, Yangling, Shaanxi Province, China) three times a day. After the acclimation period, fish (initial mean body weight, 6.36 ± 0.18 g) were randomly distributed into 12 circular 215-L tanks at a density of 12 fish per tank (*n* = 3 per diet). The tanks were supplied with recycling water and aeration to maintain enough dissolved oxygen. All rearing tanks were maintained under fluorescent lamps (12-hr light/12-hr dark). The water temperature during the trial ranged between 25 and 28°C. Each experimental diet was randomly assigned to triplicate tanks, which was fed to apparent visual satiation (three times a day, at 8:30, 12:30, and 16:30) for 8 weeks. At the initial feeding, the juvenile mirror carp run upstream to water surface to feed aggressively. After a period, the feeding intensity decreased and when all fish settled on the bottom and did not come up to feed, this state was considered as the apparent satiation. The feeding process need ensure there was no feed residue during the entire feeding process. The dissolved oxygen, pH, and ammonia content maintained at 11.6 ± 0.20 mg L^−1^, 7.28 ± 0.20, and 0.04 ± 0.01 mg L^−1^, respectively.

### 2.3. Sampling

Prior to the start of the feeding trial, six fish were sampled and stored at −20°C for proximate composition analysis. After 8 weeks, fish were starved for 24 hr prior to sampling, and then anesthetised with 0.1 g L^−1^ MS-222 (Sigma, St. Louis, MO, USA). Final body weight (FBW), body length, total length, and fish number of all fish were measured to calculate growth parameters and survival.

Blood samples (six fish per tank) were collected from the caudal vein using syringes (Zhengzhou Kangjia Medical Equipment Company, Zhengzhou Province, China). The serum was separated by centrifugation (825 g for 10 min) after 6 hr at 4°C, pooled for each tank, and frozen at −80°C. The hepatopancreas and intra-peritoneal fat (three fish per tank) were separated and stored at −80°C for quantitative polymerase chain reaction (qPCR) analyses. The hepatopancreas, intra-peritoneal fat, and muscle samples were sampled and stored at −20°C for the analysis of proximate composition (three fish per tank) and FA composition (two fish per tank). For the analysis of proximate composition, three whole fish per tank were pooled, homogenized, and stored at −20°C. Pieces measuring 0.5–1.0 cm of intra-peritoneal fat and hepatopancreas (one fish per tank) were cut and fixed in 4% formaldehyde in 1x-phosphate-buffered solution for histological analyses.

### 2.4. Proximate Composition of Diets and Fish Samples

The proximate composition of diets and tissues was analyzed by the methods recommended by the Association of Official Analytical Chemists (AOAC, 2000). Crude protein was measured by Kjeldahl method. The crude lipid and moisture were measured by ethyl ether extraction and 105°C drying to constant weight. The ash content was measured at 550°C for 4 hr with a muffle furnace (TMF-3100, EYELA Co., Tokyo, Japan). The ingredients and proximate composition of the diets are presented in Table 1.

The FA compositions (diets and tissues) were determined by gas chromatography (Agilent Technologies, Santa Clara, CA, USA) as described previously by Tian et al [17]. The results are presented as the percentage of TFA. The FA composition of the diets is presented in Table 2.

### 2.5. Determination of Serum Biochemical Parameters

The serum samples were analyzed for their content of alanine aminotransferase (ALT), aspartate transaminase (AST), total protein (TP), albumin (ALB), globulin (GLO), glucose (GLU), total cholesterol (T-cho), triglycerides (TG), high-density lipoprotein (HDL), and low-density lipoprotein (LDL) at Yangling Demonstration Area Hospital (Yangling, Shaanxi Province) with an automatic biochemical analyzer (Hitachi 7180, Tokyo, Japan).

### 2.6. Histological Observations of Hepatopancreas and Intra-Peritoneal Fat Tissue

The fixed hepatopancreas and intra-peritoneal fat samples were rinsed in running water for 24 hr. Subsequently, the samples were then dehydrated in ethanol, infiltrated in xylene, and embedded in paraffin, following standard histological procedures [18]. The samples were then cut serially (5 *μ*m thick) using a rotary microtome and stained with hematoxylin and eosin. One cross-sectional slice was prepared from each tissue sample and placed on a microscopy slide. The adipose tissue was observed and photographed using an inverted microscope (OPTEC, China). Five non-overlapping areas per piece of tissue were selected and calculated by TS View 7 (Softim aging solutions, Tucsen, China). In each area, five measurements were taken [19], resulting in a total of 25 measurements per fish. The hepatopancreas was photographed using a light microscope (Eclipse 50i, Nikon, Tokyo) and a camera (Digital Sight DS2MV with control unit DS-L2, Nikon, Tokyo).

### 2.7. RNA Extraction and Real-Time Quantitative Polymerase Chain Reaction (qRT-PCR)

Total RNA was extracted from the hepatopancreas and intra-peritoneal fat by homogenizing them in TRNzol reagent (Tiangen, Beijing, China). The extracted RNA was treated with RNase-free DNase (Ta KaRa, Dalian, China) to prevent genomic DNA amplification during reverse-transcription (RT)-PCR. RNA integrity was checked by electrophoresis on 2% agarose gels before RT-PCR. The total RNA was then reverse-transcribed into cDNA using the PrimeScript® RT reagent kit (TaKaRa, Dalian, P.R. China). Real-time qPCR was performed using a CFX 96 Real-time PCR Detection System (Bio-Rad, Hercules, CA, USA). The total volume of the PCR reaction volume was 20 *μ*L, which included 0.6 *μ*L of each primer (10 *μ*M), 1 *μ*L of the diluted cDNA, 10 *μ*L of 2xSYBR® Premix Ex TaqTMII (TaKaRa, Dalian, P.R. China), and 7.8 *μ*L of sterilized double-distilled water. The RT-PCR involved an initial activation step at 95°C for 30 s and then it was followed by 40 cycles of 95°C for 15 s and 60°C for 15 s. *β*-actin mRNA was used as the internal control. The primer sequences of genes and GenBank accession numbers are shown in Table 3. The relative expression of genes was calculated by the delta–delta CT method (2^−*ΔΔ*CT^) [20].

### 2.8. Statistical Analysis

All data are presented as mean ± S.D., and statistical analyses were performed using SPSS19.0 for Windows Software (SPSS, Chicago, IL, USA). Differences due to dietary treatments were evaluated by using two-way ANOVA (variables: lipid sources and inclusion levels) followed by Duncan's post-hoc test. For data generated from histological analyses, which had several measurements per fish, a nested analysis was performed using nesting factors area, fish, and tank [21] (R core team., 2019). Result was considered significant at *P* < 0.05. The graphs were created using Prism 7 (Graph Pad Software Inc., San Diego, USA).

Pearson correlation coefficient (PCC) was used to quantify the correlation between the tissue FA composition and dietary FA by using *R* value. The *R* value was computed using the equation: *R* = log_2_ (Pt/Pd). *R* is the degree of variation between sampled tissue FA content and diet FA, Pt is the percentage of one FA in a specific tissue, and Pd is the percentage of the FA in diets.

### 2.9. Calculations of Growth and Nutritional Indices

(1) $$\begin{array}{c} \text{Weight gain rate (WGR)}=\frac{\text{(Final weight of fish - initial weight of fish)}}{\text{Initial weight of fish}}, \end{array}$$

(2) $$\begin{array}{c} \text{Specific growth rate (SGR)}=\frac{\text{[ln (Final weight of fish) - ln (initial weight of fish)]}}{\text{Days of feeding }}\times 100\%, \end{array}$$

(3) $$\begin{array}{c} \text{Feed intake }\left({\text{FI, g fish}}^{-1}\right)=\frac{\text{Total amount of the feed consumed}}{\text{Number of fish }}, \end{array}$$

(4) $$\begin{array}{c} \text{Feed intake }\left({\text{FI, g fish}}^{-1}\;{\text{days}}^{-1}\right)=\frac{\text{Total amount of the feed consumed}}{\text{Number of fish/days }}, \end{array}$$

(5) $$\begin{array}{c} \text{Feed conversion ratio (FCR)}=\text{Feed intake}/\text{(Final weight of fish - initial weight of fish)}, \end{array}$$

(6) $$\begin{array}{c} \text{Protein efficiency ratio (PER)}=\frac{\text{(Final weight - initial weight)}}{\text{Protein intake}}\times 100\%, \end{array}$$

(7) $$\begin{array}{c} \text{Protein retention ratio (PRR)}=\frac{\text{Fish protein gain }}{\text{Protein intake}}\times 100\%, \end{array}$$

(8) $$\begin{array}{c} \text{Lipid retention ratio (LRR)}=\frac{\text{Fish lipid gain }}{\text{Lipid intake}}\times 100\%, \end{array}$$

(9) $$\begin{array}{c} \text{Survival rate (SR)}=\frac{\text{Number of final fish }}{\text{Number of initial fish }}\times 100\%, \end{array}$$

(10) $$\begin{array}{c} \text{Viscera index (VSI)}=\frac{\text{Viscera weight }}{\text{Body weight }}\times 100\%, \end{array}$$

(11) $$\begin{array}{c} \text{Condition factor (CF)}=\frac{\text{Body weight }}{{\text{Body length}}^{3}}\times 100\%, \end{array}$$

(12) $$\begin{array}{c} \text{Hepatosomatic index (HSI)}=\frac{\text{Hepatopancreas weight}}{\text{Body weight}}\times 100\%, \end{array}$$

(13) $$\begin{array}{c} \text{Intra-peritoneal fat index (IFI)}=\frac{\text{Weight of intra-peritoneal fat}}{\text{Body weight}}\times 100\%. \end{array}$$

## 3. Results

### 3.1. Growth Performance and Biological Parameters

At the end of the feeding trial, the HL group displayed significantly higher FBW, WGR, SGR, PER, PRR, and lower FCR compared to the CT group (*P* < 0.05). Both the inclusion of lipid level (HL) and its source (HL + BSFO) decreased the LRR compared to fish fed CT or CT + BSFO (*P* < 0.05). IFI was significantly influenced by lipid level and lipid source (*P* < 0.05). The FI, VSI, HSI, and CF in all groups showed no significant differences. The growth performance and biological parameters were not influenced by the interaction of lipid level and BSFO (Table 4).

### 3.2. Tissue Proximate Composition

The crude protein content of the whole body of fish fed the HL diets was significantly decreased while the crude lipid content was significantly increased compared to fish fed CT diets (*P* < 0.05). The moisture and ash content of the whole body did not show significant differences between all groups (*P* < 0.05). There were no significant differences in dietary effects on the moisture, crude protein, lipid, or ash in muscle. In the whole body, the protein content was lower in the HL fed fish compared to the two low-fat diets (*P* < 0.05). The whole body lipid was higher in the two HL diets compared to the low-lipid diets (*P* < 0.05). In the hepatopancreas, only the lipid content was affected by diets, being lowered by BSFO in the low-fat diets (*P* < 0.05), but not in the HL diets, which were both higher than the low-fat diets (Table 5).

### 3.3. Tissue FA Composition

From Tables 6, 7, and 8, we observe that the use of BSFO had a strong effect on the FA composition in the analyzed tissues (muscle, hepatopancreas, and intra-peritoneal fat). In particular, the use of BSFO resulted in the increased levels of the C12 : 0 and C14 : 0. Other effects were primarily attributed to the reduction or removal of SO, leading to a significant decrease in n-6 FA content, across all analyzed tissues in the groups that contained BSFO (*P* < 0.05). The C20 : 5n-3 (eicosapentaenoic acid) remained unaffected in all tissues, but the C22 : 6n-3 (docosahexaenoic acid, DHA) levels were higher in both groups that included BSFO. The low-fat groups exhibited the highest percentages of DHA (*P* < 0.05).

### 3.4. Relationship between the Tissue and Diet FA Composition Using PCC

It appears that the fluctuation of the *R* values is associated with both the tissues (muscle, hepatopancreas, and intra-peritoneal fat) and diets. To quantify this relationship, we calculated the PCC of FA between the different tissues and diets, as displayed in Table 9. Among the three tissues, the CT group showed the weakest correlation with the tissues, while HL + BSFO displayed the strongest correlation. The PCC varied across the tissues. The results also showed that the hepatopancreas showed the weaker correlation with diets, followed by muscle; however, the intra-peritoneal fat was highest (*P* < 0.05).

### 3.5. Relationship between Diet and FA Composition of Fish Tissue Using the R Values

**Figure 1:**
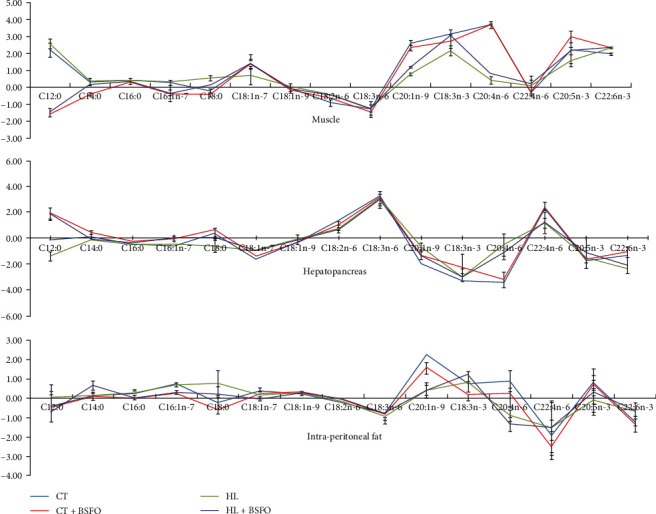
The relationship between diet and FA composition of fish tissue using the *R* values. *X*-axis represented the FAs, and *Y*-axis represented the *R* values. Values were group means, with standard deviations represented by vertical bars (*n* = 3).

If the dietary FAs are completely digested and directly deposited in the tissues, without undergoing FA metabolism (elongations, desaturations, *β*-oxidation, etc.), the fold line will merge with the *X*-axis. *R* > 0, the FA is preferentially deposited in the tissue; *R* < 0, the FA is metabolized in the tissue and/or not efficiently absorbed. In this study, all groups showed similar fluctuations in the same tissues. In muscle, the *R* values of C12 : 0, C18 : 1n-7, C18 : 3n-6, C20 : 1n-9, C18 : 3n-3, C20 : 4n-6, C20 : 5n-3, and C22 : 6n-3 were high. In hepatopancreas, the *R* values of C18 : 3n-6, C18 : 3n-3, C20 : 4n-6, and C22 : 4n-6 were high. As for intra-peritoneal fat, the *R* values of C20 : 1n-9, C20 : 4n-6, and C22 : 4n-6 were high (Figure 1).

### 3.6. Serum Biochemical Health Markers

The serum T-cho and TG content was significantly increased with higher dietary lipid levels (*P* < 0.05). However, the levels of serum ALT, AST, TP, ALB, GLO, GLU, LDL, and HDL content were not affected by the lipid levels, BSFO, or their interactions (Table 10).

### 3.7. Tissue Morphology

**Figure 2:**
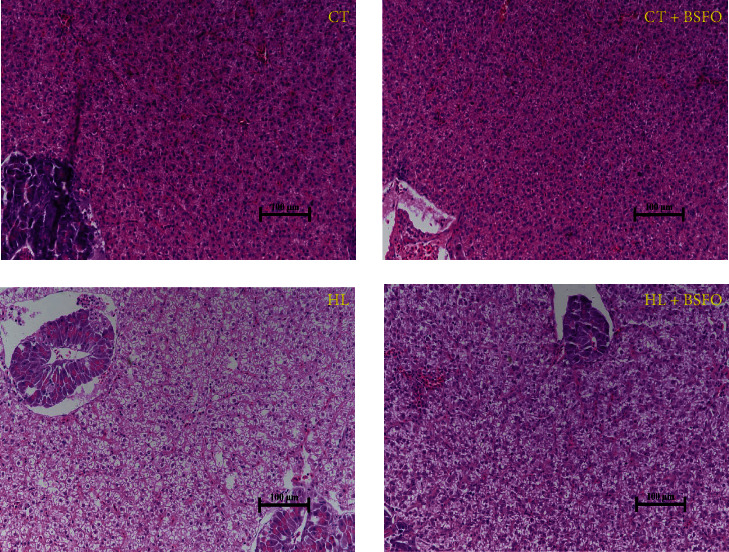
Effects of BSFO and lipid level on hepatopancreas morphology of juvenile mirror carp (20x) (*n* = 3). The descriptions of CT, CT + BSFO, HL, and HL + BSFO were the same as Table 1.

**Figure 3:**
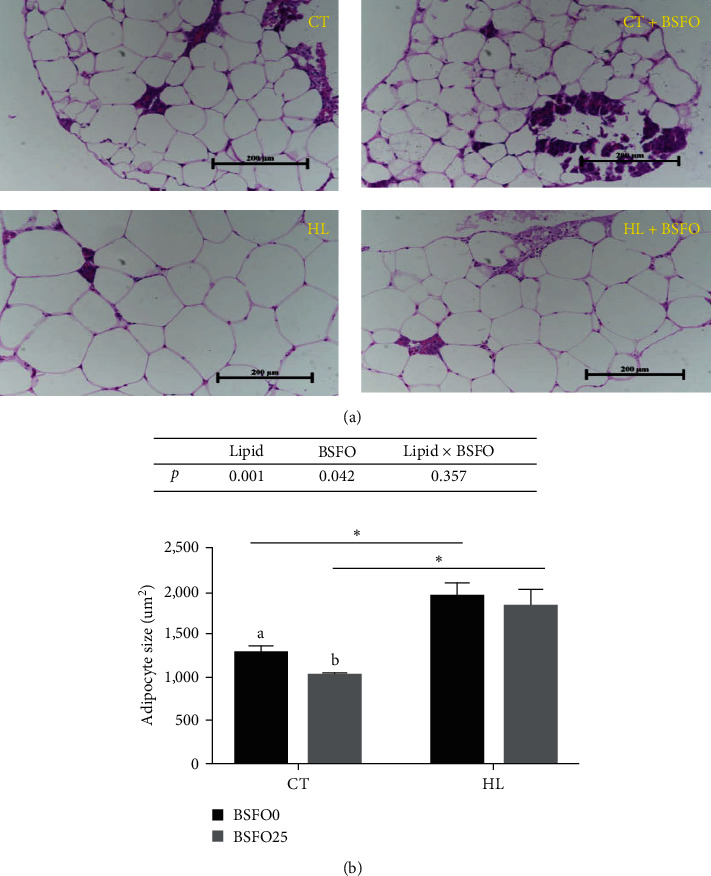
Effects of BSFO and lipid level on intra-peritoneal fat morphology of juvenile mirror carp (20x). (a) Morphological observation of adipocytes. (b) The statistics of adipocytes size; columns presented as means ± S.D. (*n* = 3). Bars bearing with different letters and symbols were significantly different among treatments (*P* < 0.05).

Figures 2 and 3 demonstrated that the hepatocytes in the CT and CT + BSFO groups showed a more rounded shape, centrally located nuclei, and clearer cell boundaries compared to the two high-fat groups. The presence of significant vacuoles in the HL fed fish indicated a greater lipid accumulation in the hepatopancreas with the higher dietary lipid levels. Moreover, the adipocytes were larger in the HL group than those in the CT group (*P* < 0.05). The adipocyte sizes were significantly reduced in the low-fat diet with BSFO group (*P* < 0.05). However, the same effect of BSFO was not observed in the HL diets. There was no influence from the interactions between lipid levels and BSFO.

### 3.8. Expression of Lipid Metabolism-Related Genes

**Figure 4:**
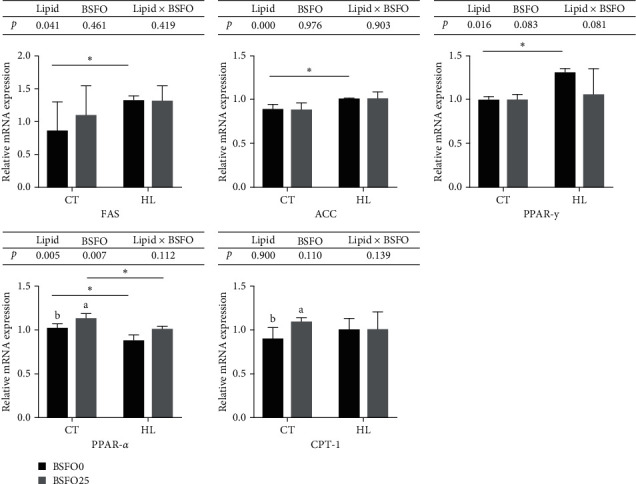
Effects of BSFO and lipid level on relative gene expression in intra-peritoneal fat of juvenile mirror carp; columns presented as means ± S.D. (*n* = 3); bars bearing with different letters and symbols were significantly different among treatments (*P* < 0.05).

**Figure 5:**
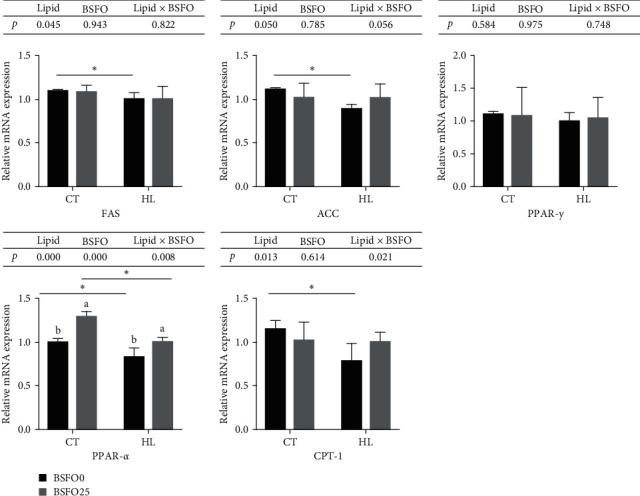
Effects of BSFO and lipid level on relative gene expression in hepatopancreas of juvenile mirror carp; columns presented as means ± S.D. (*n* = 3); bars bearing with different symbols were significantly different among treatments (*P* < 0.05).

The mRNA expression of *FAS*, *ACC*, and *PPAR-γ* in the intra-peritoneal fat tissue was significantly up-regulated in the HL fed fish compared to the diets fed low-lipid diet-fed fish (*P* < 0.05), but not affected by BSFO. However, the mRNA expression of *CPT-1* in the same fat tissue was significantly up-regulated in the low-lipid diets with BSFO. On the contrary, the hepatopancreas mRNA expressions of *FAS*, *ACC*, and *CPT-1* were significantly down-regulated in the HL fed fish compared to in the fish fed low-lipid diets (*P* < 0.05), but also not influenced by BSFO. The hepatic mRNA expression of *PPAR-γ* was not affected by the diets. Only the mRNA expression of *PPAR-α* had a similar expression pattern in both tissues, being significantly down-regulated by the HL diets (*P* < 0.05) and significantly up-regulated by BSFO in both the intra-peritoneal fat and hepatopancreas tissue (*P* < 0.05) (Figures 4 and 5).

## 4. Discussion

By increasing the dietary lipid levels, the FBW, WGR, SGR, PER, and PRR were significantly increased, while FCR significantly decreased. These findings indicated a significant promotion of growth and protein-sparing effects through increased lipid levels in the diets.

Wu et al. [22] found these similar results when using the 9% as the same lipid level for common carp. A lower LRR was observed in the HL groups, indicating that a substantial portion of these added lipids was utilized for energy supply, thus promoting the protein-sparing effect seen. This has also been previously observed in grass carp (*Ctenopharyngodon idellus*) and some other fish species when fish fed with high-lipid diets [23, 24].

Feeding high-lipid diets has been shown to increase VSI in several fish species, such as in grass carp [25], silver sillago (*Sillago sihama*) [23], cobia (*Rachycentron canadum*) [26], and Japanese seabass (*Lateolabrax japonicus*) [27]. However, in our study, we observed only a non-significant tendency toward an increased VSI in HL-fed group. Some studies have reported no increase in VSI with different lipid levels in diets. For instance, Li et al. [28] demonstrated that the VSI and HSI of large-mouth bass (*Micropterus salmoides*) were not influenced by higher dietary lipid levels between 10% and 12.5%. Similarly, a study on common carp found that lipid levels of 6%–9% in diets did not significantly influence VSI, CF, and HSI [22]. Our results suggested that the viscera did not make significant contributions to the weight gain in the HL groups, indicating potential benefits for fish health. A significant decrease in the IFI was observed in the fish fed diets with BSFO at both lipid levels. Xu et al. [13] also found a similar results in juvenile mirror carp fed a mixture of insect oils (containing 6.33 g kg^−1^ BSFO) compared to fish fed with silkworm pupa oil or YMO. This study indicated that lauric acid, as a MCFA, is known to be easily oxidized and can reduce lipid storage in fish [29–31]. The dietary MCFAs (3.24% of TFAs) in the mixture of insect oils could therefore enhance the growth of mirror carp without increasing lipid deposition. In our current trial, the BSFO diets contained 7.36% and 6.21% lauric acid of TFAs in the low- and high-lipid diets, respectively. Differently, the BSFO in the HL diets in our study did not cause lipid deposition when compared with the HL group. Upon comparison, we found that the HL + BSFO diet had a high content of C18 : 2n-6 (35.63% of TFAs, 9% lipid level). While the MIXO (YMO: BSFO: silkworm pupa oil = 1 : 1 : 1) diet used by Xu et al. [13] had a lower content of C18 : 2n-6 (30.5% of TFAs, 6% lipid level), the YMO diet had a high content of C18 : 2n-6 (33.9% of TFAs, 6% lipid level) which also lead to an increase in IFI. So, we speculated that the negative effect of high content C18 : 2n-6 maybe stronger, thereby resulting in the absence of a decrease in IFI despite the inclusion of BSFO in the diets.

The measured adipocyte sizes were larger in the HL groups than those in the fish fed the regular lipid level. Adipocyte sizes were also significantly smaller in the CT + BSFO group than that in the CT group. There was also a tendency for a smaller adipocyte size in the HL + BSFO group than in the HL group, but this was not significant. Moreover, the gene expression of *FAS*, *ACC*, and *PPAR-γ* in intra-peritoneal fat was significantly up-regulated, and the gene expression of *PPAR-α* was significantly down-regulated in the HL groups compared to the CT group. The gene expression results were consistent with the change in IFI. This indicates that high dietary lipid levels may lead to an increase in abdominal adipose tissue in mirror carp, which may mainly through the promotion of lipid synthesis and the suppression of lipid lipolysis. Similar research has been found in grass carp and Nile tilapia (*Oreochromis niloticus*) when fish are fed with high-lipid diets [24, 32]. It may also be the result of an adaptive regulatory mechanism of lipid metabolism in the abdominal adipose tissue of carp in response to the intake of a high-fat diet [33]. Subsequently, the gene expression of *PPAR-α* and *CPT-1* was significantly increased in CT + BSFO compared with that in the CT group. Similar results have been found in mirror carp fed with 25 g kg^−1^ BSFO, which is BSF fed by algae residue [34]. Our research indicated that BSFO could increase the lipid lipolysis of fish when fed with 6% lipid diets. However, 25 g kg^−1^ BSFO in high-lipid diets could not activate the gene expression of lipid lipolysis, although it showed an increasing tendency.

The study of cobia indicated that the increasing of whole body lipid content is generally correlated with dietary lipid levels [26]. In our study, the whole body lipid content also increased from 7.58 to 7.71 g kg^−1^ when the dietary lipid content changed from 6% to 9%. Similar results have been found in other fish species, including grass carp [24], European sea bass (*Dicentrarchus labrax*) [35], large-mouth bass [36], and cobia (*R. canadum*) [26]. Additionally, the crude protein content was significantly decreased when fish were fed HL diets in our study. The crude lipid content in the hepatopancreas was significantly increased, but the crude lipid content in muscle was not influenced by the dietary lipid level. This indicates that lipid accumulation in fish is tissue-biased, and lipids seem to be mainly deposited in the viscera. Similar studies on fish have indicated that a high-lipid diet increases the abdominal fat index and hepatopancreas fat content, as observed in Russian sturgeon (*Acipenser gueldenstaedtii*) [37], grass carp [24, 25], and rockfish (*Sebastes schlegelii*) [38]. It is well-known that greater fat deposition occurring in the viscera (led by high-energy diets) can reduce the commercial value of the product [39]. Therefore, this aspect must be considered when feeding fish with a high-lipid diet. In our study, the crude lipid content in the hepatopancreas was significantly decreased with the use of BSFO, regardless of the lipid diets were fed to the fish. In a previous study by our team, the author suggested that when fish are fed diets containing BSFO, there may be a faster *β*-oxidation of the dietary lipids or a more efficient transport out of the hepatopancreas to the tissues, resulting in decreased lipid content of the hepatopancreas [13]. The histomorphological observation of the hepatopancreas supports this finding. Hepatocytes in CT and CT + BSFO groups showed rounded shapes with centrally located nuclei and clear cell boundaries in CT and CT + BSFO groups compared with the other two groups. The significant presence of vacuoles in the HL group indicates a large lipid accumulation in the hepatopancreas with the increasing lipid levels. The area of vacuoles was significantly smaller in HL + BSFO than that in the HL group. It indicated that with the use of BSFO it could effectively decrease the hepatopancreas lipid content without decreasing FBW, thereby improving the commercial value of the product. Subsequently, the results of gene expression relating to the metabolism of lipids in the hepatopancreas were further examined. Our results showed that the gene expression of lipid biosynthesis (*FAS* and *ACC*) and lipid catabolism (*CPT-1* and *PPAR-α*) was significantly down-regulated in fish fed with HL diets. Similar studies about high-lipid diets suppressing lipogenesis or lipid catabolism have been observed in grass carp [24, 40], black sea bream (*Sparus macrocephalus*) [41], and blunt snout bream (*Megalobrama amblycephala*) [42]. In high-lipid diets, this effect may be due to the high content of C18 : 2n-6 in SO, which changes in the mitochondrial membrane composition, therefore decreasing the lipid catabolism gene expression [43]. The down-regulation in lipid catabolism gene expression may lead to the increased hepatopancreas lipid content. The gene expression of *PPAR-α* in the CT + BSFO and HL + BSFO groups was significantly down-regulated compared with that in the CT and HL groups. It indicated that with the use of BSFO could promote the lipid metabolism of fish and help to decrease the hepatopancreas lipid content. Some researcheres [44] found in the study on freshwater Atlantic salmon (*Salmo salar*) that fish fed with BSF diets could decrease the hepatopancreas lipid content, accompanied by the down-regulation expression of *CPT-1* and *PPAR-γ*. Moreover, some research on mammals indicated that mice fed with high-lipid diets containing MCFA could up-regulate the gene expression of *PPAR-α* when compared with a high-lipid control group [45]. Coconut oil, which is also a kind of MCFA oil, could activate *PPAR-α* in the hepatopancreas in the trained state of mice when compared to those fed with SO [46].

Based on the correlation analysis between dietary FA composition and different tissues, we observed that the tissues in the HL + BSFO group had the highest correlation with diets, followed by the HL group, CT + BSFO group, and CT group. In low-lipid diets, the proportion of lipid in low-lipid diet was higher to maintain the body's normal needs, resulting in a lower correlation with tissues. The high-lipid diet contains a higher proportion of lipids used for deposition after absorption, hence maintaining a high correlation with tissues [47]. We also found that with the use of BSFO, the correlation of diets to tissues was improved when compared with the CT and HL groups, respectively. We speculated that this improvement may be due to the function of MCFA. The role of MCFA has been widely investigated in both livestock and human nutrition due to their rapid absorption and oxidation [48]. MCFAs are more polar than other lipids and are less reliant on chylomicrons and lipoproteins for transport. Since they can be transported without the need for these systems, MCFAs can be absorbed (intact) into intestinal epithelial enterocytes and then hydrolyzed in cells by microsomal lipases [49]. Diets containing BSFO could enhance the lipid utilization, leading to more FAs being deposited in tissues, ultimately improving the correlation of FA composition between tissues and diets. On the flip side, we found that the intra-peritoneal fat tissue had the highest correlation with diets, followed by muscle and hepatopancreas. Tian et al. [50] also reported these similar results in Songpu mirror carp. Intra-peritoneal fat is the primary tissue that accumulates lipid or serves as an energy storage depot [51]. FAs from the diet are deposited here as TG or membrane phospholipids after absorption and transportation [52], which may explain the reason for its high correlation with FA composition in the diet. Hepatopancreas is an important intermediary metabolic tissue with a higher capacity for FA metabolism, such as desaturation/elongation and *β*-oxidation [53]. The FA composition of the hepatopancreas can be self-regulated and resulting in a lower correlation [54].

The absolute values of *R* represent the extent of variation in FAs [50]. Considering the influence of dietary treatments, the variation trends of *R* were similar within a particular tissue, indicating the similar metabolism patterns of the FAs in the same tissue. There was clear tissue-specificity regarding FA composition. The C18 : 2n-6 was considered as the essential FA for carp [52]. It should be noticed that the proportions of C18 : 2n-6 in the hepatopancreas (14.89%–25.03%) were relatively lower than those in IPF and muscle. The IPF showed the highest content (25.62%–35.26%). The intra-peritoneal fat showed the lowest C20 : 4n-6 (0.28%–0.70%) and DHA (0.50%–0.62%) contents among the tissues, and the highest C18 : 2n-6 contents, suggesting that the intra-peritoneal fat of mirror carp might prefer to retain C18 polyunsaturated fatty acids (PUFAs) but not HUFAs compared to other tissues, similar to our earlier studies in grass carp [54] and Songpu mirror carp [50]. This indicated a tissue-specific manner of deposition for the retention of these FAs. FAs can affect the flavor and nutritional properties of muscles, and DHA deposition in muscles, as an edible part, helps to meet human nutritional needs. Our results indicated that with the use of BSFO, the DHA content was significantly increased in CT + BSFO and HL + BSFO groups. Our results align with the research of Li et al. [14], which found that feeding Jian carp with diets containing 25 g kg^−1^ could improve the DHA content in muscle.

## 5. Conclusion

This study indicated that a high-lipid level resulted in an increase in feed utilization, protein utilization, and growth performance of juvenile mirror carp. Diets with 25 g kg^−1^ BSFO contained in 60 g kg^−1^ lipid level diets could decrease the IFI, adipocyte size, and the hepatopancreas lipid content, while also up-regulating of lipolysis-related gene expression without negatively impacting growth performance. However, the effectiveness of lipid metabolism for BSFO contained in 90 g kg^−1^ lipid level diets was not as pronounced as in 60 g kg^−1^ lipid level diets. Moreover, diets containing BSFO could improve the content of total n-3 PUFA, while reducing the content of total n-6 PUFA in the muscle.

## Figures and Tables

**Table 1:** Ingredients and proximate composition of the experimental diets.

Ingredients (g kg^−1)^	CT	CT + BSFO	HL	HL + BSFO
Fish meal	100	100	100	100
Soybean meal	140	140	140	140
Rapeseed meal	210	210	210	210
Cottonseed meal	230	230	230	230
Full fat soybean meal	40	40	40	40
Rice bran	120	120	120	120
Flour	85	85	50	50
BSFO	0	25	0	25
Soybean oil	25	0	60	35
Ca(H_2_PO_4_)_2_	20	20	20	20
Bentonite	20	20	20	20
Premix	10	10	10	10
Feed composition				
Moisture	89.1	90.8	90.8	89.1
Crude protein	325.6	325.5	325.0	325.1
Crude lipid	60.4	60.7	93.1	93.3
Ash	96.4	96.6	97.0	96.5

Premix ingredients including (/kg): vitamin A 4,000 IU, vitamin D_3_ 800 IU, vitamin E 50 IU, vitamin B_1_ 2.5 mg, vitamin B_2_ 9 mg, vitamin B_6_ 10 mg, vitamin C 250 mg, nicotinic acid 40 mg, pantothenic acid, calcium 30 mg, biotin 100 *μ*g, betaine 1,000 mg, Fe 140 mg, Cu 2.5 mg, Zn 65 mg, Mn 19 mg, Mg 230 mg, Co 0.1 mg, I 0.25 mg, Se 0.2 mg; the CT presented diet containing lipid level 60 g kg^−1^ and only using SO as oil source; the CT + BSFO presented diet containing lipid level 60 g kg^−1^ and only using BSFO as oil source; the HL presented diet containing lipid level 90 g kg^−1^ and only using SO as oil source; the HL + BSFO presented diet containing lipid level 90 g kg^−1^ and accompanied by BSFO used as part of oil source.

**Table 2:** FA composition (% of TFAs) of diets fed to juvenile mirror carp (*Cyprinus carpio* var. specularis).

Parameter	Diet			
	CT	CT + BSFO	HL	HL + BSFO
C12 : 0	0.21	7.36	0.13	6.21
C14 : 0	1.06	2.72	0.82	1.54
C16 : 0	13.71	16.48	11.81	13.91
C18 : 0	0.40	0.56	0.21	0.29
∑SFA	15.39	27.13	12.98	21.95
C16 : 1n-7	1.53	3.42	1.06	2.30
C18 : 1n-7	3.37	3.04	3.58	3.22
C18 : 1n-9	29.88	29.87	25.57	25.66
C20 : 1n-9	0.31	0.42	1.51	1.22
∑MUFA	35.09	36.76	31.72	32.40
C18 : 2n-6	40.81	28.37	44.15	35.63
C18 : 3n-6	5.74	4.65	7.68	6.59
C20 : 4n-6	0.18	0.24	1.37	1.31
C22 : 4n-6	0.87	0.97	0.52	0.66
∑n-6PUFA	47.60	34.22	53.72	44.19
C18 : 3n-3	0.29	0.41	0.43	0.29
C20 : 5n-3	0.21	0.16	0.28	0.24
C22 : 6n-3	1.42	1.32	0.86	0.92
∑n-3PUFA	1.92	1.89	1.58	1.45
∑PUFA	49.52	36.11	55.30	45.64
n-3/n-6 PUFA	4.03	5.52	2.94	3.28

*Notes*: SFA = Saturated fatty acid; MUFA = Monounsaturated fatty acids; PUFA = Polyunsaturated fatty acids. The descriptions of CT, CT + BSFO, HL, and HL + BSFO were the same as Table 1.

**Table 3:** Primers used for qPCR analysis.

Genes	Forward (5–3)	Reverse (5–3)	Accession number	Size (bp)
*FAS*	TGCTGGATGCTTTGTTTGAG	ACTACACCACCAGCGATTCC	KY378913.1	85
*PPAR-γ*	CTTCGTGAACCTGGACTTG	ATCTGACCGTAGGAGATGAG	XM_019096045.1	123
*PPAR-α*	CATGTCCCACA ACGCTATC	TGTCTGAGGTAGGCTTCAT	FJ849065.1	156
*CPT-1*	CAGATGGAAAGTGTTGCTAATGAC	TGTGTAGAAGTTGCTGTTGACCA	JF728839	172
*ß-actin*	GCAGATGTGGATTAGCAAGCAG	TTGAGTCGGCGTGAAGTGG	M24113.1	100

*Notes*: *FAS = Fatty acid synthase; PPAR-γ = Peroxisome proliferator-activated receptor-γ; PPAR-α = Peroxisome proliferator-activated receptor-α; CPT-1 = Carnitine palmitoyltransferase−1*.

**Table 4:** Effects of BSFO and lipid level on growth performance and biological parameters of juvenile mirror carp (*n* = 3).

Parameters	Diets				ANOVA, *Pr > F*		
	CT	CT + BSFO	HL	HL + BSFO	Lipid	BSFO	Lipid × BSFO
IBW (g)	6.36 ± 0.01	6.36 ± 0.02	6.36 ± 0.01	6.36 ± 0.02	0.488	0.072	0.587
FBW (g)	66.27 ± 1.27^b^	65.20 ± 1.93^b^	69.45 ± 1.09^a^	67.30 ± 0.89^ab^	0.010	0.072	0.506
WGR (%)	9.42 ± 0.20^b^	9.25 ± 0.31^b^	9.92 ± 0.17^a^	9.57 ± 0.14^ab^	0.010	0.074	0.501
SGR (%/d)	4.18 ± 0.03^b^	4.16 ± 0.06^b^	4.27 ± 0.03^a^	4.21 ± 0.02^ab^	0.010	0.095	0.533
FCR	1.32 ± 0.02^a^	1.32 ± 0.03^a^	1.26 ± 0.02^b^	1.29 ± 0.01^ab^	0.005	0.128	0.349
FI (g/fish)	78.83 ± 0.76	78.05 ± 0.85	79.21 ± 0.53	78.69 ± 0.40	0.216	0.128	0.749
FI (%BW/d)	1.40 ± 0.01	1.39 ± 0.02	1.41 ± 0.01	1.41 ± 0.01	0.216	0.301	0.747
PER (%)	233.69 ± 4.31^b^	231.68 ± 5.15^b^	244.99 ± 2.95^a^	238.10 ± 2.49^ab^	0.004	0.082	0.307
PRR (%)	30.11 ± 0.53^b^	29.80 ± 0.72^b^	31.23 ± 0.40^a^	30.44 ± 0.20^ab^	0.015	0.089	0.429
LRR (%)	101.63 ± 1.83^a^	99.86 ± 1.80^a^	70.16 ± 0.44^b^	68.09 ± 0.69^b^	0.000	0.039	0.850
VSI (%)	8.90 ± 0.49	9.04 ± 0.75	9.34 ± 1.16	9.41 ± 0.99	0.448	0.848	0.945
HSI (%)	1.94 ± 0.10	1.93 ± 0.11	1.92 ± 0.03	1.93 ± 0.04	0.831	0.987	0.715
IFI (%)	0.47 ± 0.01^b^	0.43 ± 0.01^c^	0.50 ± 0.02^a^	0.49 ± 0.01^ab^	0.001	0.014	0.219
CF	3.27 ± 0.05	3.26 ± 0.08	3.27 ± 0.06	3.26 ± 0.05	0.923	0.704	0.970

*Notes*: Values are means ± S.D. Values in the same line with different superscript letters are significantly different (*P* < 0.05). IBW = Initial body weight; FBW = Final body weight; WGR = Weight gain rate; SGR = Special growth rate; FCR = Feed conversion ratio; FI = Feeding intake; PER = Protein retention ratio; PRR = Protein retention ratio; LRR = Lipid retention ratio; VSI = Viscera index; HSI = Hepatosomatic index; IFI = Intra-peritoneal fat index; CF = Conditional factor. The descriptions of CT, CT + BSFO, HL, and HL + BSFO were the same as Table 1.

**Table 5:** Effects of BSFO and lipid level on body composition of juvenile mirror carp (% wet weight, *n* = 3).

Parameters	Diets				ANOVA, *Pr > F*		
	CT	CT + BSFO	HL	HL + BSFO	Lipid	BSFO	Lipid × BSFO
Whole body							
Moisture	74.98 ± 0.02	74.94 ± 0.38	74.58 ± 0.30	74.19 ± 0.40	0.093	0.523	0.617
Crude protein	13.19 ± 0.02^a^	13.20 ± 0.05^a^	13.05 ± 0.09^b^	13.09 ± 0.18^ab^	0.012	0.618	0.604
Crude lipid	7.58 ± 0.10^b^	7.54 ± 0.01^b^	7.71 ± 0.01^a^	7.71 ± 0.02^a^	0.000	0.536	0.630
Ash	2.65 ± 0.15	2.62 ± 0.23	2.66 ± 0.29	2.63 ± 0.19	0.907	0.738	0.946
Muscle							
Moisture	78.51 ± 0.50	78.94 ± 0.45	78.62 ± 0.94	78.65 ± 0.75	0.709	0.320	0.386
Crude protein	17.35 ± 0.79	17.82 ± 0.70	17.83 ± 0.25	17.53 ± 0.60	0.718	0.762	0.145
Crude lipid	1.0.4 ± 0.01	1.04 ± 0.02	1.03 ± 0.02	1.04 ± 0.02	0.150	0.423	0.432
Ash	1.26 ± 0.02	1.24 ± 0.02	1.25 ± 0.02	1.26 ± 0.03	0.633	0.338	0.193
Hepatopancreas							
Moisture	78.38 ± 0.61	78.48 ± 0.46	78.09 ± 0.64	78.13 ± 0.40	0.079	0.672	0876
Crude protein	12.77 ± 0.40	13.15 ± 1.46	12.84 ± 1.45	12.18 ± 1.26	0.379	0.780	0.307
Crude lipid	6.70 ± 0.15^b^	6.28 ± 0.18^c^	6.93 ± 0.12^a^	6.85 ± 0.18^a^	0.000	0.000	0.010
Ash	1.22 ± 0.07	1.33 ± 0.11	1.28 ± 0.14	1.25 ± 0.09	0.773	0.336	0.110

*Notes*: Values are means ± S.D. Values in the same line with different superscript letters are significantly different (*P* < 0.05). The descriptions of CT, CT + BSFO, HL, and HL + BSFO were the same as Table 1.

**Table 6:** Effects of BSFO and lipid level on muscle FA composition of juvenile mirror carp (% TFA, *n* = 3).

Muscle	Groups				ANOVA, *Pr >F*		
	CT	CT + BSFO	HL	HL + BSFO	Lipid	BSFO	Lipid × BSFO
C12 : 0	1.03 ± 0.36^b^	2.40 ± 0.22^a^	0.79 ± 0.43^b^	2.36 ± 0.55^a^	0.244	0.000	0.945
C14 : 0	1.35 ± 0.25^b^	2.03 ± 0.19^a^	1.07 ± 0.31^b^	1.74 ± 0.09^a^	0.004	0.000	0.728
C16 : 0	18.57 ± 1.34^ab^	20.07 ± 1.35^a^	16.12 ± 2.13^b^	17.70 ± 2.12^ab^	0.005	0.030	0.803
C18 : 0	0.35 ± 0.04^b^	0.43 ± 0.05^a^	0.30 ± 0.07^b^	0.32 ± 0.04^b^	0.003	0.040	0.18
∑SFA	21.31 ± 1.93^b^	24.93 ± 1.25^a^	18.29 ± 3.01^c^	22.11 ± 1.83^ab^	0.003	0.001	0.877
C16 : 1n-7	1.88 ± 0.22^b^	2.53 ± 0.73^a^	1.32 ± 0.09^b^	1.89 ± 0.38^b^	0.018	0.020	0.545
C18 : 1n-7	8.77 ± 1.50	9.28 ± 2.85	6.01 ± 3.09	8.26 ± 2.91	0.183	0.299	0.262
C18 : 1n-9	28.88 ± 3.26	26.70 ± 4.37	27.10 ± 3.4	25.23 ± 3.15	0.211	0.243	0.985
C20 : 1n-9	1.90 ± 0.22^b^	2.17 ± 0.54^b^	2.57 ± 0.23^a^	2.78 ± 0.08^a^	0.000	0.076	0.9
∑MUFA	41.43 ± 2.89^a^	40.67 ± 2.70^a^	37.00 ± 0.84^b^	38.16 ± 0.97^b^	0.015	0.848	0.394
C18 : 2n-6	22.60 ± 2.45^b^	18.12 ± 1.68^c^	31.94 ± 3.71^a^	25.80 ± 2.57^b^	0.000	0.002	0.283
C18 : 3n-6	2.53 ± 0.86^ab^	1.70 ± 0.41^b^	3.25 ± 0.57^a^	2.81 ± 0.73^a^	0.009	0.055	0.586
C20 : 4n-6	2.39 ± 0.08^b^	3.03 ± 0.57^a^	1.83 ± 0.36^b^	2.26 ± 0.10^bc^	0.001	0.004	0.374
C22 : 4n-6	0.68 ± 0.10	0.78 ± 0.14	0.56 ± 0.18	0.73 ± 0.30	0.475	0.124	0.56
∑n-6PUFA	28.20 ± 3.04^b^	23.62 ± 1.79^c^	37.58 ± 3.99^a^	31.60 ± 2.99^b^	0.002	0.005	0.393
C18 : 3n-3	2.58 ± 0.43	2.93 ± 0.88	1.97 ± 0.79	2.30 ± 1.09	0.088	0.416	0.717
C20 : 5n-3	0.99 ± 0.19	1.28 ± 0.40	0.85 ± 0.23	1.08 ± 0.65	0.540	0.181	0.914
C22 : 6n-3	5.49 ± 0.29^b^	6.57 ± 0.31^a^	4.30 ± 0.17^d^	4.75 ± 0.11^c^	0.000	0.000	0.031
∑n-3PUFA	9.06 ± 0.78^b^	10.78 ± 1.39^a^	7.12 ± 0.99^c^	8.12 ± 1.60^bc^	0.000	0.026	0.708
∑PUFA	37.26 ± 2.54^bc^	34.40 ± 2.21^c^	44.71 ± 3.19^a^	39.73 ± 1.78^b^	0.000	0.019	0.299
n-3/n-6PUFA	0.33 ± 0.06^b^	0.46 ± 0.07^a^	0.19 ± 0.05^c^	0.26 ± 0.07^bc^	0.082	0.007	0.492

*Notes*: Values are means ± S.D. Values in the same line with different superscript letters are significantly different (*P* < 0.05). The descriptions of CT, CT + BSFO, HL, and HL + BSFO were the same as Table 1.

**Table 7:** Effects of BSFO and lipid level on hepatopancreas FA composition of juvenile mirror carp (% TFA, *n* = 3).

Hepatopancreas	Groups				ANOVA, *Pr > F*		
	CT	CT + BSFO	HL	HL + BSFO	Lipid	BSFO	Lipid × BSFO
C12 : 0	0.26 ± 0.14^b^	2.17 ± 0.70^a^	0.34 ± 0.17^b^	1.97 ± 0.46^a^	0.812	0.001	0.506
C14 : 0	0.97 ± 0.10^c^	2.10 ± 0.25^a^	0.93 ± 0.08^c^	1.69 ± 0.23^b^	0.022	0.000	0.054
C16 : 0	19.04 ± 2.20	19.52 ± 1.62	17.69 ± 3.04	18.18 ± 2.36	0.478	0.857	0.913
C18 : 0	0.32 ± 0.03^ab^	0.36 ± 0.05^a^	0.31 ± 0.09^ab^	0.28 ± 0.01^b^	0.519	0.828	0.229
∑SFA	20.60 ± 2.39^ab^	24.17 ± 1.39^a^	19.28 ± 3.27^b^	22.12 ± 1.97^ab^	0.343	0.059	0.846
C16 : 1n-7	2.44 ± 0.45^b^	3.59 ± 0.17^a^	1.50 ± 0.21^c^	2.35 ± 0.29^b^	0.000	0.00l	0.187
C18 : 1n-7	10.64 ± 1.40^a^	8.05 ± 1.47^b^	7.13 ± 1.42^b^	6.20 ± 1.38^b^	0.002	0.013	0.092
C18 : 1n-9	37.75 ± 5.13^a^	39.51 ± 2.63^a^	29.73 ± 5.48^b^	30.39 ± 2.76^b^	0.003	0.465	0.942
C20 : 1n-9	1.32 ± 0.18^b^	1.14 ± 0.33^b^	2.53 ± 0.59^a^	3.00 ± 0.87^a^	0.002	0.411	0.21
∑MUFA	52.14 ± 5.56^a^	52.29 ± 3.24^a^	40.89 ± 5.24^b^	41.95 ± 2.14^b^	0.001	0.559	0.648
C18 : 2n-6	16.71 ± 1.76^b^	14.89 ± 3.32^b^	27.59 ± 4.09^a^	25.03 ± 4.39^a^	0.001	0.206	0.65
C18 : 3n-6	0.72 ± 0.26^ab^	0.55 ± 0.19^b^	0.95 ± 0.21^a^	0.83 ± 0.32^ab^	0.085	0.668	0.967
C20 : 4n-6	2.06 ± 0.73	2.16 ± 1.06	2.13 ± 1.04	2.87 ± 0.65	0.483	0.299	0.634
C22 : 4n-6	0.19 ± 0.05	0.18 ± 0.10	0.23 ± 0.13	0.28 ± 0.10	0.11	0.807	0.838
∑n-6PUFA	19.68 ± 2.24^b^	17.78 ± 2.65^b^	30.91 ± 4.55^a^	29.01 ± 3.51^a^	0.000	0.257	0.72
C18 : 3n-3	2.83 ± 1.05	2.54 ± 1.23	3.66 ± 0.80	2.52 ± 1.09	0.477	0.155	0.758
C20 : 5n-3	0.79 ± 0.22	0.52 ± 0.18	0.77 ± 0.42	0.56 ± 0.39	0.573	0.266	0.944
C22 : 6n-3	3.94 ± 2.33	2.71 ± 0.70	4.50 ± 2.94	3.83 ± 2.16	0.282	0.468	0.979
∑n-3PUFA	7.57 ± 2.46	5.77 ± 1.07	8.93 ± 2.95	6.91 ± 2.40	0.171	0.152	0.865
∑PUFA	27.26 ± 4.22^b^	23.55 ± 3.54^b^	39.84 ± 4.50^a^	35.93 ± 2.40^b^	0.000	0.059	0.656
n-3/n-6PUFA	0.38 ± 0.10	0.32 ± 0.04	0.30 ± 0.11	0.25 ± 0.12	0.35	0.349	0.814

*Notes*: Values are means ± S.D. Values in the same line with different superscript letters are significantly different (*P* < 0.05). The descriptions of CT, CT + BSFO, HL, and HL + BSFO were the same as Table 1.

**Table 8:** Effects of BSFO and lipid level on intra-peritoneal fat FA composition of juvenile mirror carp (% TFA, *n* = 3).

Intra-peritoneal fat	Groups				ANOVA, *Pr >F*		
	CT	CT + BSFO	HL	HL + BSFO	Lipid	BSFO	Lipid × BSFO
C12 : 0	0.16 ± 0.12^c^	5.30 ± 0.55^a^	0.16 ± 0.05^c^	3.89 ± 0.13^b^	0.000	0.000	0.000
C14 : 0	1.11 ± 0.14^c^	2.91 ± 0.15^a^	0.91 ± 0.11^c^	2.42 ± 0.64^b^	0.031	0.000	0.424
C16 : 0	15.93 ± 1.10^a^	16.20 ± 0.91^a^	14.08 ± 1.37^b^	14.09 ± 0.53^b^	0.012	0.917	0.775
C18 : 0	0.33 ± 0.06	0.38 ± 0.06	0.41 ± 0.13	0.34 ± 0.09	0.938	0.950	0.593
∑SFA	17.53 ± 1.33^c^	24.79 ± 0.90^a^	15.56 ± 1.39^d^	20.75 ± 0.67^b^	0.001	0.000	0.479
C16 : 1n-7	2.53 ± 0.09^c^	4.04 ± 0.12^a^	1.68 ± 0.18^d^	2.84 ± 0.25^b^	0.000	0.000	0.029
C18 : 1n-7	4.41 ± 0.51^a^	3.56 ± 0.48^b^	4.03 ± 0.40^a^	3.10 ± 0.25^b^	0.111	0.005	0.836
C18 : 1n-9	35.28 ± 1.73^a^	36.49 ± 1.56^a^	30.79 ± 0.85^b^	30.83 ± 1.07^b^	0.000	0.326	0.229
C20 : 1n-9	1.49 ± 0.06^ab^	1.24 ± 0.76^b^	2.03 ± 0.37^a^	1.66 ± 0.49^ab^	0.047	0.206	0.585
∑MUFA	43.71 ± 2.19^ab^	45.33 ± 2.42^a^	38.52 ± 0.99^b^	38.42 ± 0.71^b^	0.000	0.296	0.158
C18 : 2n-6	33.34 ± 1.92^b^	25.62 ± 1.33^c^	39.26 ± 1.78^a^	34.54 ± 0.73^b^	0.000	0.000	0.055
C18 : 3n-6	3.29 ± 0.24^ab^	2.60 ± 1.30^b^	4.06 ± 0.42^a^	3.82 ± 0.68^a^	0.015	0.212	0.368
C20 : 4n-6	0.38 ± 0.12^bc^	0.28 ± 0.12^c^	0.70 ± 0.13^a^	0.52 ± 0.12^b^	0.006	0.136	0.321
C22 : 4n-6	0.23 ± 0.04	0.17 ± 0.06	0.28 ± 0.24	0.30 ± 0.26	0.521	0.989	0.672
∑n-6PUFA	37.24 ± 2.17^b^	28.67 ± 2.52^c^	44.30 ± 2.04^a^	39.18 ± 1.00^b^	0.000	0.000	0.065
C18 : 3n-3	0.51 ± 0.19^b^	0.45 ± 0.11^b^	0.76 ± 0.03^a^	0.67 ± 0.12^a^	0.003	0.343	0.608
C20 : 5n-3	0.39 ± 0.16	0.27 ± 0.06	0.28 ± 0.06	0.36 ± 0.21	0.868	0.893	0.192
C22 : 6n-3	0.62 ± 0.17	0.50 ± 0.20	0.58 ± 0.30	0.61 ± 0.21	0.901	0.961	0.479
∑n-3PUFA	1.52 ± 0.46	1.21 ± 0.23^c^	1.62 ± 0.35	1.64 ± 0.36	0.157	0.651	0.410
∑PUFA	38.76 ± 2.25^b^	29.88 ± 2.46^c^	45.92 ± 2.24^a^	40.83 ± 1.03^b^	0.000	0.000	0.060
n-3/n-6PUFA	0.04 ± 0.02	0.04 ± 0.01	0.04 ± 0.01	0.04 ± 0.01	0.458	0.228	0.984

*Notes*: Values are means ± S.D. Values in the same line with different superscript letters are significantly different (*P* < 0.05). The descriptions of CT, CT + BSFO, HL, and HL + BSFO were the same as Table 1.

**Table 9:** PCC analysis of the relationship between the tissue and diet FA composition.

Parameters	Diets				ANOVA, *Pr> F*		
	CT	CT + BSFO	HL	HL + BSFO	Lipid	BSFO	Lipid × BSFO
Muscle	0.90 ± 0.04^bA^	0.94 ± 0.01^abA^	0.95 ± 0.01^aA^	0.96 ± 0.01^aAB^	0.007	0.065	0.141
Hepatopancreas	0.76 ± 0.06^aB^	0.86 ± 0.03^bB^	0.91 ± 0.04^bB^	0.91 ± 0.05^bB^	0.018	0.307	0.184
Intra-peritoneal fat	0.96 ± 0.01^A^	0.96 ± 0.01^A^	0.97 ± 0.01^A^	0.98 ± 0.01^A^	0.061	0.337	0.664
ANOVA, *Pr > F*							
Diets	0.000						
Tissue	0.000						
Diets × tissue	0.02						

*Notes*: Values are means ± S.D. Values in the same line with different lowercase letters are significantly different (*P* < 0.05). Values in the same column with different capital letters are significantly different (*P* < 0.05). The descriptions of CT, CT + BSFO, HL, and HL + BSFO were the same as Table 1.

**Table 10:** Effects of BSFO and lipid level on serum indexes of juvenile mirror carp (*n* = 3).

Parameters	Diets				ANOVA, *Pr > F*		
	CT	CT + BSFO	HL	HL + BSFO	Lipid	BSFO	Lipid × BSFO
ALT (U/L)	53.33 ± 2.08	54.67 ± 3.06	55.00 ± 1.00	56.00 ± 2.00	0.263	0.377	0.897
AST (U/L)	139.0 ± 3.0	138.33 ± 4.51	139.67 ± 2.52	138.67 ± 1.53	0.789	0.652	0.928
TP (g/l)	54.67 ± 1.15	53.33 ± 1.53	54.00 ± 1.00	54.00 ± 1.00	0.899	0.360	0.360
ALB (g/L)	13.00 ± 0.10	13.00 ± 1.00	13.00 ± 1.00	13.33 ± 0.58	0.715	0.715	0.715
GLO (g/L)	46.17 ± 1.15	40.0 ± 1.73	40.33 ± 1.15	41.0 ± 1.73	0.849	0.573	0.207
GLU (mmol/L)	7.0 ± 1.0	7.67 ± 1.53	8.0 ± 1.0	8.33 ± 1.53	0.296	0.521	0.828
T-cho (mmol/L)	2.63 ± 0.15^b^	2.57 ± 0.15^b^	2.90 ± 0.10^a^	2.85 ± 0.05^a^	0.004	0.430	0.908
TG (mmol/L)	3.17 ± 0.21^b^	3.37 ± 0.15^b^	4.17 ± 0.31^a^	3.90 ± 0.10^a^	0.000	0.787	0.086
HDL (mmol/L)	2.40 ± 0.10	2.27 ± 0.15	2.30 ± 0.10	2.37 ± 0.06	0.446	0.796	0.446
LDL (mmol/L)	1.25 ± 0.13	1.24 ± 0.05	1.27 ± 0.15	1.23 ± 0.06	0.979	0.779	0.816

*Notes*: Values are means ± S.D. Values in the same line with different superscript letters are significantly different (*P* < 0.05). ALT = Alanine aminotransferase; AST = Aspartate transaminase; TP = Total protein; ALB = Albumin; GLO = Globulin; GLU = Glucose; T-cho = Total cholesterol; TG = Total glycerol; HDL = High-density lipoprotein; LDL = Low-density lipoprotein; SOD = Superoxide dismutase; CAT = Catalase; MDA = Maleic dialdehyde. The descriptions of CT, CT + BSFO, HL, and HL + BSFO were the same as Table 1.

## Data Availability

Data will be available from the first author by reasonable request.
